# Exploring the moderating effects of SIRT1 and gene polymorphisms rs7895833 on the relationship between hemoglobin levels and physical frailty in elderly adults with comorbid chronic diseases: A moderated mediation analysis

**DOI:** 10.12688/f1000research.133517.3

**Published:** 2024-05-08

**Authors:** Dedi Ardinata, Novita Sari Harahap, Nenni Dwi Aprianti Lubis, Tetty Aman Nasution

**Affiliations:** 1 Department of Physiology, Faculty of Medicine, Universitas Sumatera Utara, Medan, North Sumatra, Indonesia; 2 Department of Sport Science, Faculty of Sport Science, Universitas Negeri Medan, Medan, North Sumatra, Indonesia; 3 Department of Nutrition, Faculty of Medicine, Universitas Sumatera Utara, Medan, North Sumatra, Indonesia; 4 Department of Microbiology, Universitas Sumatera Utara, Medan, North Sumatra, Indonesia

**Keywords:** Hemoglobin, physical frailty, SIRT1 expression, SNP rs7895833, moderated mediation analysis.

## Abstract

**Background:**

Relationship age, hemoglobin, and physical frailty have all been investigated in older people with more than one chronic disease. There has been little analysis of the relationship between hemoglobin, age, physical frailty, plasma levels of Sirtuin1 (SIRT1), and the gene polymorphism (SNP) rs7895833 A>G. The goal of this study was to find out how SIRT1 level, SNP rs7895833, hemoglobin, age, and physical frailty (frail score) are related in older Indonesian adults with comorbid chronic diseases.

**Methods:**

This was an observational study. Demographic and clinical data were retrieved from the electronic health records of Universitas Sumatera Utara Hospital, Medan, Indonesia. Physical frailty, SIRT1 level, and SNP rs7895833 were measured using an appropriate and valid method. Purposive sampling was used to determine the eligibility of 132 elderly adults from November 2022 to February 2023.

**Results:**

The indirect effect of hemoglobin on the frail score (FS) through age was negative and significant, according to a conditional mediation analysis (β=-0.0731; p=0.023). Meanwhile, the direct effect of hemoglobin on the FS was negative and not significant (β=0.1632; p=0.052). According to the conditional moderated mediation analysis, the size of the direct effect of age on FS was increased by genotype AG-GG and SIRT1 level (β _low_=0.2647; p=0.002, β _middle_=0.2956; p<0.001, and β _high_=0.319; p<0.001). The size of the conditional indirect effect of Hemoglobin on FS through age was negative and significantly increased by SNP genotype AG-GG and SIRT1 level (β _low_=-0.0647; p=0.032, β _middle_=-0.0723; p=0.024, and β _high_=-0.078; p=0.02).

**Conclusions:**

Higher plasma levels of SIRT1 and the SNP genotype AG-GG may both contribute to physical frailty in the elderly population. Hemoglobin levels in the blood fall with age, which can negatively impact older persons who already have chronic diseases. However, the interactions between these factors are intricate, requiring more study to completely understand the processes underlying development.

## Introduction

Frailty has a yearly incidence rate of 4.34%, with a global prevalence rate of 13.6% among older adults. ^1^ In Indonesia, however, the number of people aged 60 and older was 18.1 million in 2010, representing 7.6% and is projected to rise to 33.7 million by 2025 and 48.2 million by 2035. ^2^

Among older adult residents, it has been shown that the prevalence of multimorbidity is between 55 and 98%, anemia was within 19 and 76%, and frailty is around 70 and 93%. ^3^^–^^5^ According to the literature, older adults who have more chronic diseases are more vulnerable to the risk developing anemia. ^6^ Anemia and frailty in older adults were found to be correlated, according to a systematic review and meta-analysis of observational studies. ^7^ Some cross-sectional studies indicate a relationship between older adults’ frailty and hemoglobin levels. ^8^^,^^9^ The following hypothesis is therefore proposed: Hypothesis 1 (H1): Hemoglobin is negatively related to FS.

The aging process or the presence of comorbidities are associated to the development of frailty. ^10^ Frailty and age-related chronic diseases are not only often associated, but one increases the risk of the other, suggesting a bidirectional association between frailty and comorbidity aging-related disorders. ^11^^,^^12^ The pathobiology of aging relates chronic disease, multimorbidity, and frailty, and this knowledge provides criteria for diagnosis and management approaches. ^13^ Able to follow is the hypothesis: Hypothesis 2 (H2): Age is positively related to FS.

Hemoglobin is a protein found in red blood cells that carries oxygen throughout the body. ^14^ Low hemoglobin levels, known as anemia, are a common condition in older adults and can be caused by a variety of factors, including nutritional deficiencies, chronic diseases, and medications ^15^ which can lead to a decrease in physical function and an increased risk of frailty. Age is a major risk factor for physical frailty ^16^^,^^17^ which is defined as a state of decreased physiological reserve and increased vulnerability to stressors. ^18^ As people age, they may experience declines in muscle mass and strength, balance and coordination, and cardiovascular and respiratory function, which can contribute to physical frailty. ^19^ Based on the preceding considerations, the following hypothesis is proposed: Hypothesis 3 (H3): Age mediates the relationship between hemoglobin and FS.

Previous studies have indicated that the SIRT1 SNP rs7895833 is located in the promoter region of the SIRT1 gene, about 21kb upstream of the coding region. ^20^ Its location in the promoter suggests that it could influence SIRT1 gene expression levels. Altered SIRT1 levels caused by the SNP could dysregulate these processes and lead to much worse health outcomes, including chronic obstructive pulmonary disease, ^21^ cardiovascular diseases, ^22^ oxidative stress, ^23^ essential hypertension and type 2 diabetes mellitus, ^24^ coronary artery disease, ^25^ rheumatoid arthritis, ^26^ dyslipidaemia, ^27^ metabolic syndrome, ^28^ rheumatoid arthritis, ^29^ and neurodegenerative disease. ^30^ There was limited research on the specific interaction between SIRT1 SNP rs7895833, hemoglobin, age, and physical frailty. However, it is possible that these factors may be related to each other through various biological pathways.

SIRT1 has been related to both frailty and deteriorating health outcomes. However, other investigations have produced inconsistent results. Using Fried’s criteria, there was no consistent correlation between SIRT1 and frailty in older people living in the community, ^31^ and there was no correlation between frailty and serum-induced SIRT1 expression. ^32^ Participants in the lowest quintile had a lower likelihood of being weak, but serum-induced SIRT1 expression was not related to age or mortality. ^33^ Kumar *et al.*, found frail people’s serum sirtuin levels (SIRT1 and SIRT3) were much lower than those of non-frail persons. ^34^ Frail older adults had higher levels of SIRT1 than robust older adults. ^35^

Even though the relationship of age, hemoglobin, and frailty in older adults has been studied, there is currently limited studies on the association of these three variables with plasma levels of SIRT1 and the SNP genotype rs7895833 in elderly adults with chronic disease comorbidity. As a consequence, we come to the following hypothesis: Hypothesis 4 (H4): SNP genotype AG-GG and plasma levels of SIRT1 negatively moderates the effect of hemoglobin on FS through age as a mediator.

The purpose of this study was to investigate the effects of plasma levels of SIRT1 and the SIRT1 SNP rs7895833 A>G in the promoter region on the relationship between hemoglobin, age, and frailty in Indonesian older adults with chronic diseases comorbid.

## Methods

This study follows ‘A Guideline for Reporting Mediation Analyses of Randomized Trials and Observational Studies (AGReMA) ^36^ and The Strengthening the Reporting of Observational studies in Epidemiology (STROBE). ^37^

We conducted observational (cohort) studies of the moderated mediation analyses to find any potential causal effects. The mediator role of age in the association between hemoglobin level and frail scale score is explained by plasma levels of SIRT1 and the NSP rs7895833 genotype serving as moderating variables.

The study included 132 older adults who were scheduled to receive outpatient care at the Universitas Sumatera Utara Hospital in Medan, Indonesia, and who met the study criteria: men and women over the age of 60 with a complete electronic health record of laboratory and clinical data, as well as having chronic diseases one year prior. There are no mental or physical disorders that are interfering with their capacity to respond to questionnaire questions. A purposive sampling approach was used to choose the study subjects. The sample size was determined using the following formula ^38^:

$$n=\frac{{Z_{1-\alpha ∕2}}^{2}\;p\left(1-p\right)}{d^{2}}$$

Z _1-α/2_^2^/22 (type I error 5%)=1.96, expected proportion in population based on previous studies (p)=18.7%, ^39^ absolute error/precision (d)=0.05.

### Measurements

*Sociodemographic, clinical, and laboratory data*

We used sociodemographic, clinical, and laboratory data results from an electronic health record database after clinical diagnoses were determined for each subject one year ago.

*Frailty assessment*

The physical frailty phenotype was assessed that used the frailty scale score. During face-to-face interviews with the patients, trained nursing staff used the Indonesian version of the FRAIL scale, which has been proven to be a reliable and valid screening tool for frailty syndrome assessment. ^40^ The FRAIL scale (FS) consists of five components: fatigue, resistance, ambulation, sickness, and weight loss. The FRAIL scale scores range from 0 to 5 (one point for each component; 0 represents best to 5 represents worst) and indicate robust (0), pre-frail (1 to 2), frail (3 to 5). The physical frailty phenotype was also treated as a continuous variable, ranging from 0 to 5, in the study.

*Blood sampling*

Blood samples were collected from each subject in line with approved protocols. Subjects were instructed to fast for at least 8 hours before blood collection, which took place between 8:00 and 10:00 a.m. Professional phlebotomists collected 2 mL of blood in tubes containing EDTA using a predefined method.

*Blood processing and DNA isolation*

The collected blood samples were immediately processed for plasma separation. Plasma samples were aliquoted and stored at -80°C until analysis. Genomic DNA was extracted from the residual white blood cells using a DNA isolation kit (Wizard ^®^, Madison, USA, A1120) and the manufacturer’s instructions. All pure DNA samples were kept at 2-8°C until PCR was used for SIRT1 genotyping.

*SIRT1 assay*

The plasma levels of SIRT1 was determined by a monoclonal antibody-based ELISA method using a commercially available human SIRT1 ELISA kit (Elabscience ^®^, Houston, USA, E-EL-H1546). Microtiter plates were coated with an antibody specific to human SIRT1. 100 μL standard and plasma samples were pipetted into the appropriate wells, and the protocol was followed by using a secondary antibody and then avidin conjugated horseradish peroxidase. The formation of horseradish peroxidase was measured using ELISA reader (Thermo Fisher Scientific, Finland) at 450 nm. Seven different concentrations of purified SIRT1 (20, 10, 5, 2.500, 1.250, 0.630, and 0.31 ng/mL) were used to plot a standard curve. The inter- and intra-assay coefficients of variation were 4.04% and 4.55% respectively, with a detection range of 0.31–20 ng/mL.

*Determination of SIRT1 gene polymorphisms*

The single nucleotide polymorphism (SNP) rs7895833 was selected because it is one of the most frequent polymorphisms in the SIRT1 gene, and has been associated frailty and several diseases.

SIRT1 rs7895833 A>G in the promoter region ^41^ were analysed using polymerase chain reactions with two-pair primers (PCR-CTPP) assay with minor modifications. ^42^ The SIRT1 gene encompassing rs7895833 A>G, polymorphic sites were amplified by PCR using the primers. ^41^ Briefly, 25 ml total PCR mixtures containing 100–200 ng DNA, 10.0 pmol of each primers, 1.0 mM dNTP (deoxynucleotide triphosphates), 25 mM MgCl2 and 2.5U Taq DNA polymerase in the supplied reaction buffer (Taq Buffer with (NH _4_) _2_SO _4_) were prepared. PCR was performed with the primers were the following:

Forward primer 1: CCCAGGGTTCAACAAATCTATGTTG

Forward primer 2: CCCAGGGTTCAACAAATCTATGTTG

Reverse primer 1: GCTTCCTAATCTCCATTACGTTGAC

**Figure 1.**
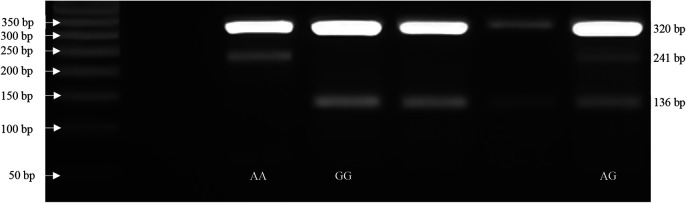
SIRT1 SNP rs7895833 A>G representative PCR gel electrophoresis images.

Reverse primer 2: CCTCCCAGTCAACGACTTTATC

with the initial denaturation at 95°C for 10 min.; 30 cycles of 95°C for 1 min., 64°C for rs7895833 A>G polymorphism, PCR products were visualized on a 2% agarose gel with ethidium bromide staining and genotyped. The genotypes for polymorphism were defined by 3 distinct banding patterns ( Figure 1); for rs7895833 AG polymorphism: 320, 241 bp for AA genotype; 320, 241 and 136 bp for AG genotype; and 320, 136 bp for GG genotype. ^41^

### Statistical evaluation

Hardy–Weinberg equilibrium tests were performed with Hardy-Weinberg Equilibrium online calculator.

All statistical analyses were carried out using statistical tool jamovi ver.2.3. and the significance was set at p<0.05 (two-tail). Descriptive statistics were used to examine all of the subject’s characteristics. Normally distributed variables were reported using means and standard deviations, while non-normally distributed ones were summarized with medians and ranges. Qualitative variables were described using the numbers of events and frequencies.

The correlation plot (Qgraph) created with jamovi’s seolmatrix. This module was used to determine how the potential major variables interacted with one another. ^43^

The jAMM GLM Mediation Model was used to analysis the conditional process. The conditional process analysis to determine how much more the mechanism(s) by which an effect operates is conditional on or varies depending on the nature, environment, stimulus, or individual variations. ^44^ Mediation and moderation could be integrated analytically as a conditional process model in a variety of ways, depending on which step of the mediation process is moderated, the number of mediators, the number of moderators, and whether or not the direct effect is likewise moderated. ^45^

*Mediating effect*

An analysis of mediation was investigated to assess if age mediated the relation between hemoglobin level and frail score. This model was used to determine if hemoglobin and frail score had a considerable indirect influence. When mediator variables are included, the direct effect is diminished but still statistically significant; this effect is known as “partial mediation”. Complete mediation shows that the direct effect is no longer significant when mediator variables have been included. ^46^

*Moderating effect*

A subgroup evaluation was carried in the multiple-mediation model to see whether there was a moderating effect on simple pathways. It was considered that the existence of the moderation effect in this path was shown by the statistically significant difference in the path coefficient between the two variables. ^47^

*Moderated mediation*

Testing for mediation effects in each subgroup will lead in a biased estimated parameter and low statistical power, in accordance with Edwards’ hypothesis. The estimated parameters, including the total, indirect, and direct effects of the moderated mediation model, were conducted by integrating moderation and mediation models. Subgrouping analysis was solely used to test which path the moderator affected. ^48^^,^^49^

Before testing all mediational hypotheses, conditional process analysis depicting all interactions was developed using the general linear mediation model (i.e., the GLM mediation model) with factor coding “dummies” for genotypes AA=0 and AG-GG = 1 and covariates scaling “standardized” for hemoglobin level and plasma levels of SIRT1. A standard (delta method) procedure that leverages the approximation from the central limit theorem ^50^ was used to test the significance of the total and indirect effects, and the coefficient confidence intervals were deemed statistically significant if they did not include zero.

### Ethical considerations

This study was authorized by the Universitas Sumatera Utara Health Ethical Committee under the number 1097/KEPK/USU/2022. Each participant completed a structured questionnaire after signing informed consent, which included questions about their demographics, level of physical frailty (Frail scale), and willingness to submit a blood sample. The Declaration of Helsinki ethical principles were followed during the study’s conduction.

## Results

### Hardy–Weinberg equilibrium tests

The genotypic frequencies of the SNP rs7895833 A>G in the SIRT1 gene were not in accordance with those expected by Hardy-Weinberg equilibrium in the current investigation, confirming a previous study. ^51^ We looked at several possibilities: first, we evaluated the possibility of recruiting bias. Second, faults in the real-time PCR genotyping experiment. In each batch, however, proper controls were applied, and the probes used were definitely capable of discriminating between genotypes. Third, the Hardy-Weinberg departure occurred by chance since the observed and expected genotype frequency variations were minimal.

### Characteristics of the study’s subjects

This study included 132 eligible subjects. More than half of the participants were female (51.5%). The median age was 65 (range: 60 to 85), and the FS was 1 (range: 0 to 3). Meanwhile, the mean hemoglobin level was 12.35 (±1.8) mg/dL, and the plasma SIRT1 level was 57.1 (±32.3) ng/ml. Table 1 shows the study subjects’ detailed characteristics.

**Table 1.** Demographic, clinical and genotype characteristics of elderly adults with multimorbid (n=132).

Characteristics		Characteristics	
Age (year)	65 (60-85) ^a^	Comorbidities	
Sex (Female)	68 (51.5) ^b^	Diabetes mellitus	82 (62.12) ^b^
Frail score	1 (0-3) ^a^	Cardiovascular disease	81 (61.36) ^b^
%BMI loss 1 year	2.06 (-12.2 – 24.6) ^a^	Hypertension	58 (43.94) ^b^
Sirtuin1 plasma level (ng/ml)	57.1 (32.3) ^c^	Arthritis	49 (37.12) ^b^
Systolic Blood Pressure (mmHg)	138.5 (21.7) ^c^	Stroke	35 (26.52) ^b^
Diastolic Blood Pressure (mmHg)	75.13 (10.81) ^c^	Chronic lung disease	23 (17.42) ^b^
*Hemoglobin* (g/dL)	12.35 (1.8) ^c^	Chronic kidney disease	17 (12.88) ^b^
Fasting blood glucose (mg/dL)	120 (83 – 420) ^a^	Cancer	7 (5.30) ^b^
HbA1c (%)	7.95 (2.49) ^c^	Genotype rs7895833	
Total cholesterol (mg/dL)	195.18 (43.89) ^c^	AA	16 (12.1) ^b^
Triglyceride (mg/dL)	132 (53 – 669) ^a^	AG	79 (59.8) ^b^
High-density lipoprotein (mg/dL)	45 (20-445) ^a^	GG	37 (28.0) ^b^
Low-density lipoprotein (mg/dL)	133.74 (42.19) ^c^	AG-GG	116 (87.9) ^b^
Blood urea nitrogen (mg/dL)	20.3 (5.9 – 114.5) ^a^	AA-AG	95 (72.0) ^b^
Creatinine (mg/dL)	0.9 (0.1 – 15.1) ^a^	Allele rs7895833	
		A	111 (42.05) ^b^
		G	153 (57.95) ^b^

^**a**^ Median (min–max).

^**b**^ n (%).

^**c**^ Mean (±SD).

### The interrelationship between the potential main variables

**Figure 2.**
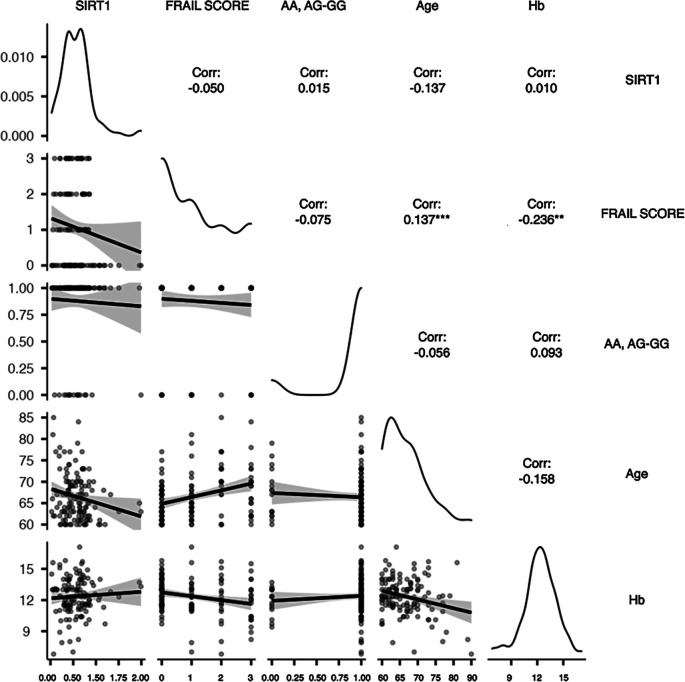
The correlation plot of the interrelationship between the potential main variables. Hb (Hemoglobin); SIRT1 (Sirtuin1); Frail score (frailty); genotype (AA, AG-GG). Spearman's rank correlation with two tail significant: *p<0.05, **p<0.01, ***p<0.001.

There was a negative relationship between hemoglobin levels and FS (r=-0.236, p=0.006). The age yielded similar results (r=-0.259, p=0.003). Meanwhile, age was also shown to have a significant positive association with FS (r=0.325, p=0.006) but a negative correlation with SIRT1 (r=-0.192, p=0.027). There were no statistically significant correlations or relationships detected between the SNP genotypes AA, AG-GG and any of the covariates ( Figure 2).

### Hypothesis testing

According to Baron and Kenny’s ^47^ criterion, there are three conditions for the existence of mediating role: 1) there is a significant correlation between independent variable (hemoglobin) and dependent variable (physical frailty); 2) there is a significant correlation between independent variable (hemoglobin) and mediating variables (age); 3) finally, the regression coefficients of independent variables (hemoglobin) and mediating variable (age) are simultaneously regressed to the dependent variable (physical frailty), the coefficient of mediator should be significant and the coefficient of independent variable become non-significant (complete mediation) or reduced (partial mediation).

Figure 2 depicts them in accordance with the partial correlation’s results. It can be seen that hemoglobin and FS had a moderately strong negative relationship (r=-0.236, p=0.006), and that age and FS had a moderately strong positive relationship (r=0.325, p<0.001). Therefore, the first and second conditions are satisfied. Hence, H1 and H2 were accepted.

**The mediating effect of age**

**Figure 3.**
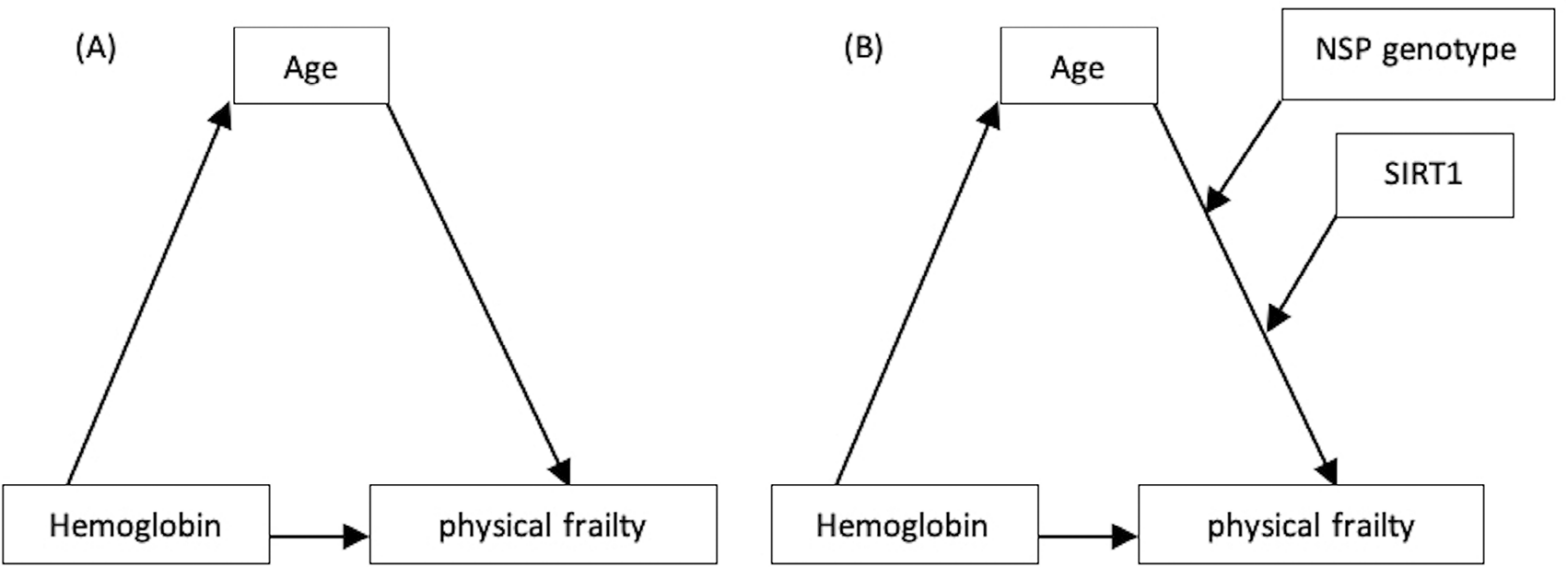
A conceptual diagram that shows hypothesized interactions between variables study; (A) Mediation model, (B) Moderated mediation model with two moderators.

Mediation analyses for direct effects, indirect mediation by age, and total effects are provided in Figure 3A and Table 2. When age was included in the model, the following occurred: The direct effect of hemoglobin on FS was negative and non-significant (β=-0.1632, p=0.052). The indirect effect or indirect mediation of hemoglobin on FS was negative and significant (β=-0.0731, p=0.023) and the total effect was negative and significant (β=-0.2363, p=0.005). As a consequence, we may conclude that age completely mediates the negative relationship between hemoglobin and FS, with a significant model mediation effect of 13% (R ^2^=0.13, p=0.001, F=9.68). As a result, H3 was accepted. This result reveals that lower hemoglobin levels may lead to physical frailty and that this effect was totally influenced by the increasing age of an elderly adult with comorbid chronic diseases.

**Table 2.** The mediation effect of age between hemoglobin and frail score.

Age	*ab*	*a*	*b*	*c’*	*c*
	-0.0731; 0.023	-0.2587; 0.002	0.2828; <0.001	-0.1632; 0.052	-0.2363; 0.005
Full model effect	R ^2^	F	p		
	0.13	3.43	0.004		

n=132. ***ab**=*mediated or indirect effect of hemoglobin on frail score through age, ***a*** =path from hemoglobin to age, ***b*** =path from age to frail score (controlling for hemoglobin), ***c'*** =direct effect from hemoglobin to frail score (controlling for age), ***c*** =total effect of hemoglobin on frail score (ab + c’). Confidence intervals computed with method: Standard (Delta method). Betas are completely standardized effect sizes. Value expreting on β; *p.*

**Moderated mediation relationship**

The moderated mediation model was then put to the test. The SNP genotypes (AA and AG-GG) and SIRT1 were integrated into this model ( Figure 3B and Table 3). Conditional moderated mediation was assessed at levels of SIRT1 of 25% (low), 50% (middle), and 75% (high) and SNP genotypes of AA and AG-GG. We noted the following: The size of the conditional direct effect of age on FS was moderately positive and significantly increased by SNP genotypes AA and SIRT1 level (β _middle_=0.1828, p=0.034 and β _high_=0.2087, p=0.015). Whereas by genotype AG-GG and SIRT1 level (β _low_=0.2647, p=0.002, β _middle_=0.2956, p<0.001, and β _high_=0.319, p<0.001). The size of the conditional direct effect of hemoglobin on FS was moderately negative but non-significant by SNP genotypes AA, AG-GG, and all SIRT1 levels. The size of the conditional indirect effect of hemoglobin on FS through age was moderately negative and significantly increased by SNP genotypes AG-GG and SIRT1 level (β _low_=0.2647, p=0.002, β _middle_=0.2956, p<0.001, and β _high_=0.319, p<0.001). The total effect was negative and significant (β=-0.2246, p=0.008). such that the conditional negative indirect effect of hemoglobin on FS completely through age becomes stronger when the SNP genotype was AG-GG and levels of SIRT1 increase (moderated mediation). The SNP genotype and SIRT1 have a 14.1% moderated mediation effect (R ^2^=0.141, p=0.004, F=3.43) Therefore, H4 was accepted. The study may have found that individuals with the AG-GG genotype and higher levels of SIRT1 may experience a stronger negative indirect effect of hemoglobin on physical frailty, compared to individuals with the AA genotype and lower levels of SIRT1. According to these analyses, decreased hemoglobin levels can lead to physical frailty, and this effect is completely mediated by age, through the SNP genotype AG-GG and elevated SIRT1 levels in elderly adults with comorbid chronic diseases.

**Table 3.** The moderation effects of SNP genotypes AA, AG-GG, and SIRT1 between hemoglobin and frail score through age as mediation variables.

Genotype * SIRT1	*ab*	*a*	*b*	*c’*	*c*
AA * 25%	-0.0364; 0.137	-0.2444: 0.003	0.149; 0.085	-0.1633; 0.063	-0.2246: 0.008
AA * 50%	-0.0447; 0.085	-0.2444: 0.003	0.1828; 0.034	-0.163; 0.064	-0.2246: 0.008
AA * 75%	-0.051; 0.061	-0.2444: 0.003	0.2087; 0.015	-0.1625; 0.064	-0.2246: 0.008
AG-GG * 25%	-0.0647; 0.032	-0.2444: 0.003	0.2647; 0.002	-0.1608; 0.055	-0.2246: 0.008
AG-GG * 50%	-0.0723; 0.024	-0.2444: 0.003	0.2956; <0.001	-0.1594; 0.058	-0.2246: 0.008
AG-GG * 75%	-0.078; 0.02	-0.2444: 0.003	0.319; <0.001	-0.1581; 0.061	-0.2246: 0.008
Full model effect	R ^2^	F	p		
	0.141	3.43	0.004		

n=132. ***ab**=*mediated or indirect effect of hemoglobin on frail score through age, ***a*** =path from hemoglobin to age, ***b*** =path from age to frail score (moderated by genotype and SIRT1, and controlling for hemoglobin), ***c'*** =direct effect from hemoglobin to frail score (controlling for age), ***c*** =total effect of hemoglobin on frail score (ab+c’). Confidence intervals computed with method: Standard (Delta method). Betas are completely standardized effect sizes. Value expreting on β; *p.*

## Discussion

In terms of physical frailty, the current research was conducted in a moderated-moderated mediation or conditional moderated mediation relationship. Hypotheses have been created based on the conceptual model in Figure 1 and the findings of prior studies. The findings supported each of the research hypotheses. The model provided the five variables that supported our conceptual model.

A recent study revealed that the relationship between low hemoglobin levels and physical frailty in older adults with one or more chronic diseases was affected by the age.

Age is a major risk factor for physical frailty, ^16^^,^^17^ which is defined as a state of decreased physiological reserve and increased vulnerability to stressors. ^18^ As people age, they may experience declines in muscle mass and strength, balance and coordination, and cardiovascular and respiratory function, which can contribute to physical frailty. ^19^

Low hemoglobin levels, also called anemia, is a condition in which the number of red blood cells or the amount of hemoglobin in the blood drops. This usually happens in older adults. Causes of anemia in older adults include nutritional deficiency, chronic kidney disease, chronic inflammation, and occult blood loss from gastrointestinal malignancy, although in many patients the aetiology were unknown. ^8^^,^^52^^–^^54^ Previous studies have shown that the prevalence of physical frailty increases with age, and that low hemoglobin levels are also more common in older adults. ^53^^,^^55^ In addition, there is evidence to suggest that low hemoglobin levels are a risk factor for physical frailty, as anemia can lead to reduced physical function and decreased muscle mass.

According to the study, low hemoglobin levels and physical frailty were related. Age completely mediated the effect of low hemoglobin levels on physical frailty, indicating that the relationship between these two variables was only significant when age was excluded. This study further showed that age completely mediated this effect and that SNP genotype AG-GG and high SIRT1 levels further moderated this effect in elderly adults with comorbid chronic diseases. This showed that the following two factors, including the existence of comorbid chronic diseases and a specific genetic variant (AG-GG) of SNP SIRT1, further moderated the relation between low hemoglobin levels and physical frailty. The correlation between low hemoglobin levels and physical frailty, in particular, may have depended on the levels of SIRT1, a protein involved in cellular metabolism, among older adults with comorbid chronic diseases and the AG-GG genotype.

There was a complex interaction between hemoglobin levels, physical frailty, age, and genetic factors that could have big effects on older people with chronic conditions. The blood contained the important protein hemoglobin, which was responsible for carrying oxygen from the lungs to the body’s tissues and organs. ^56^ Decreased hemoglobin levels can lead to anemia, which can cause fatigue, weakness, and physical frailty.

Physical frailty is a common concern for elderly people, and it is a complex condition affected by several factors, including age, chronic diseases, life styles, and heredity. Studies have shown that elderly adults, particularly those with chronic diseases, who have low hemoglobin levels are more plausible to become physically frail. ^57^^–^^59^

Furthermore, genetic factors may contribute to the development of physical frailty in elderly adults. The SIRT1 SNP rs7895833 A>G is closely associated with an increased risk of developing chronic diseases., such as cardiovascular disease, ^22^^,^^25^ chronic obstructive pulmonary disease, ^21^ Parkinson’s disease, ^60^ type 2 diabetes mellitus, ^61^ rheumatoid arthritis. ^29^ These chronic diseases can contribute to the development of physical frailty in elderly adults by reducing muscle mass, strength, and function ^11^^,^^62^ and also anemia. ^63^

SIRT1, a protein that protects cells from oxidative stress, controls glucose and lipid metabolism, and increases DNA integrity by binding to and deacetylating numerous substrates, may also have a role in physical frailty. ^64^^,^^65^ SIRT1 is regarded to be one of the potential molecules for promoting healthy aging because of its protective activities against numerous age-related diseases. Some studies have suggested that SIRT1 levels decrease with age; according to several studies, SIRT1 has been studied for its potential role in aging and age-related diseases. ^23^^,^^66^^,^^67^ Additionally, it is well known that it alters skeletal muscle metabolism and function, two major considerations in physical frailty.

There were few studies on the role of SIRT1 in iron metabolism related anaemia of chronic disease or anaemia of inflammation that decreases plasma iron with suppression of erythropoiesis. A study showed that activating SIRT1 can reduce iron accumulation in splenic macrophages by inhibiting hepcidin. ^68^ This suggests that SIRT1 may inhibit ferroptosis by reducing intracellular iron levels. Another study showed that Intestinal SIRT1 deficiency improved iron metabolism in ethanol-induced liver injury in mice, reducing ferroptosis in hepatocytes. ^69^

There are still not many studies that show what role the SNP rs7895833 A>G, especially genotype AG-GG, plays in the activity of SIRT1 in older adults. The patients with the wild-type (AA) genotype had a significantly higher level of SIRT1 protein and a significantly lower level of endothelial nitric oxide synthase (eNOS) expression. Patients with the heterozygote mutant (AG) genotype also had a significantly higher level of SIRT1 protein, but eNOS expression was not significantly different. ^22^ Kilic *et al.*, who observed that older people carrying both the wild-type (AA) genotype and the heterozygote (AG) genotype had significantly higher SIRT1 expression levels in plasma, while older people carrying the heterozygote mutant (AG) genotype had a significantly higher SIRT1 level compared with older people carrying the wild-type (AA) genotype. ^23^

As was already mentioned, the NSP rs7895833 A>G affects the expression of the SIRT1 gene. It can impact the expression of the SIRT1 gene because it is located in the promoter region, 21 kb upstream of the gene. Thus, the activity of SIRT1 appears to be regulated by a promoter-dependent change in expression mechanism, which may have an impact on the elderly’s metabolism or the progression of neurological disease. Chromatin and transcription are regulated by SIRT1, linking NAD+ signaling and metabolism to the regulation of cellular activities. ^70^

### Limitations

This study has established moderated mediation analysis among the drivers of physical frailty in elderly adults with comorbid chronic diseases, but it is not without limitations. Some limitations of the present study should be noted: The study used an observational (cohort) design and studied only elderly individuals from a university hospital, which may constitute a selection bias, meaning that we may not have been able to establish a causal relationship between the variables of interest. Another constraint of the study is the sample size, which was insufficient to detect all associations with the other studied variables and could limit the generalizability of the results. Other factors, such as multi-comorbidity, BMI, lifestyle habits (including routine exercise, diet, etc.), environmental factors, and medications, could have influenced the results, and we may not have accounted for these variables. However, our findings are supported by previously published literature; several studies with probabilistic samples and a greater number of individuals reported changes in physical frailty in relation to hemoglobin, age, the polymorphism rs7895833, involving genotype AG-GG, and plasma SIRT1 levels.

## Conclusion

The present study emphasizes the nuanced interaction of biological variables, especially SIRT1 plasma levels, NSP rs7895833 genotype, and age, in influencing physical frailty in elderly people with chronic diseases. Our findings suggests that SIRT1 levels and genotype modify the effect of age on frailty.

## Data Availability

Figshare: Exploring the moderating effects of SIRT1 protein expression and gene polymorphisms rs7895833 on the relationship between hemoglobin levels and physical frailty in elderly adults with comorbid chronic diseases. A moderated mediation analysis, https://doi.org/10.6084/m9.figshare.22492603.v2. ^71^ Data are available under the terms of the Creative Commons Attribution 4.0 International license (CC-BY 4.0).
