# Supplementary material for: Exploring the moderating effects of SIRT1 and gene polymorphisms rs7895833 on the relationship between hemoglobin levels and physical frailty in elderly adults with comorbid chronic diseases: A moderated mediation analysis
Source: F1000Res. 2024 May 8;12:510. Originally published 2023 May 17. [Version 3] doi: 10.12688/f1000research.133517.3 (PMC11066533; doi:10.12688/f1000research.133517.3)
Supplement: Peer review history

# Reviewer report: Reviewer response for version 3

**DOI:** 10.5256/f1000research.165990.r266845

**Published:** 2024-06-06

**Authors:** Amit Singh

**Affiliations:** 1 LMBI, National Institute on Aging, Baltimore, Maryland, USA

Authors have answered all my concerns, Manuscript can be approved.

Is the work clearly and accurately presented and does it cite the current literature?

Yes

If applicable, is the statistical analysis and its interpretation appropriate?

Yes

Are all the source data underlying the results available to ensure full reproducibility?

Yes

Is the study design appropriate and is the work technically sound?

Partly

Are the conclusions drawn adequately supported by the results?

Partly

Are sufficient details of methods and analysis provided to allow replication by others?

Yes

Reviewer Expertise:

Molecular Biology, Immunology and Aging

I confirm that I have read this submission and believe that I have an appropriate level of expertise to confirm that it is of an acceptable scientific standard.

# Reviewer report: Reviewer response for version 2

**DOI:** 10.5256/f1000research.164941.r253713

**Published:** 2024-05-02

**Authors:** Made Putra Semadhi

**Affiliations:** 1 Padjadjaran University, Sumedang, Indonesia; 2 Prodia National Reference Laboratory, Jakarta, Indonesia

For the methodology,

 1. It would be clearer if the author divided the subject into each comorbid/chronic disease. Because the influence of previous clinical conditions/chronic diseases cannot be completely ruled out and may interfere with the results. Diabetes, Kidney Disease, Liver Disease, Hypertension, Sarcopenia, Muscle and bone problems etc. can have different impacts on the subject. It is recommended for the author to expand the relationship analysis, explanation and discussion through this clinical condition.

 2. For better analysis, authors should ensure that all subjects are in homogeneous BMI groups, or if subjects have different BMI scores, it would be better if those BMI scores are used as analytical groupings. Previous studies have shown that BMI may have a correlation with plasma SIRT1 levels and that this would impact the results.

 3. Please add some information about SNP rs7895833 A>G in the background as well as in the discussion (pathological pathway/cascade). It would be interesting if the author could include broader information regarding the biological impacts associated with this SNP also on population distribution in Indonesia/North Sumatra.

 4. It would be better if the author could provide or explore more explanations about the subject's activities (routine exercise, eating/diet, etc.) apart from assessing the weakness score. This activity may interfere with plasma SIRT1 levels as a critical variable.

 5. In addition to completing number 4, the author must ensure that blood sampling for SIRT1 in all subjects is carried out under homogeneous conditions

 6. The conclusions drawn will be broader following additional updated explanations

Is the work clearly and accurately presented and does it cite the current literature?

Yes

If applicable, is the statistical analysis and its interpretation appropriate?

Yes

Are all the source data underlying the results available to ensure full reproducibility?

Partly

Is the study design appropriate and is the work technically sound?

Partly

Are the conclusions drawn adequately supported by the results?

Partly

Are sufficient details of methods and analysis provided to allow replication by others?

Partly

Reviewer Expertise:

Biochemistry and Geriatrics

I confirm that I have read this submission and believe that I have an appropriate level of expertise to confirm that it is of an acceptable scientific standard, however I have significant reservations, as outlined above.

# Reviewer report: Reviewer response for version 1

**DOI:** 10.5256/f1000research.146512.r253712

**Published:** 2024-04-04

**Authors:** Amit Singh

**Affiliations:** 1 LMBI, National Institute on Aging, Baltimore, Maryland, USA

Report for Ardinata et al.- Exploring the moderating effects of SIRT1 protein expression and gene polymorphisms rs7895833 on the relationship between hemoglobin levels and physical frailty in elderly adults with comorbid chronic diseases: A moderated mediation analysis.

 Ardinata et al claim to study the effects of SIRT1 protein expression and the associated SIRT1 promoter SNP in Indonesian older adult cohort. They further correlated these levels with hemoglobin, age, and frailty with chronic diseases comorbid. Study emphasize the known SIRT1 effects in interesting cohort and will be useful for aging related studies.

 Study lacks in following areas and should be updated accordingly.

 Authors claim SIRT1 levels and its relationship with various parameters. It should be corrected in title and also in text that these are plasma levels of SIRT1 not cellular protein. Explanation of plasma source of SIRT1 should be provided.

 Manuscript holds just one data figure of genotyping gel. In genotyping gel image the marker lane is labelled as 50bp ladder, first, the marker bands are not clear and second if we follow the marker bands (which should be labelled for each band) the size doesn’t matches the genotype product DNA bands'. The 136bp according to the marker will be 75bp and 241bp will be 200bp and so on **.** Please lable the gel properly.

 Since, SIRT1 is major focus of the manuscript, authors are encouraged to show at least some correlation plots of all 132 donors with SIRT1 levels and frailty or other important parameter.

 Repetitions from introduction in discussion part should be avoided. Last paragraph of discussion has many typographical errors and there are also such errors thought out the manuscript, such mistakes should be avoided.

 Conclusions can be shortened and many extreme speculations can be avoided.

Is the work clearly and accurately presented and does it cite the current literature?

Yes

If applicable, is the statistical analysis and its interpretation appropriate?

Yes

Are all the source data underlying the results available to ensure full reproducibility?

Yes

Is the study design appropriate and is the work technically sound?

Partly

Are the conclusions drawn adequately supported by the results?

Partly

Are sufficient details of methods and analysis provided to allow replication by others?

Yes

Reviewer Expertise:

Immunology, Aging, Inflammation

I confirm that I have read this submission and believe that I have an appropriate level of expertise to confirm that it is of an acceptable scientific standard, however I have significant reservations, as outlined above.

# Response

**Published:** 2024-04-11

**Authors:** Dedi Ardinata

**Affiliations:** Universitas Sumatera Utara, and Department of Physiology, Faculty of Medicine, Medan, Indonesia

Dear Amit Singh

 Thank you for your thorough review and valuable suggestions regarding our manuscript entitled "Exploring the moderating effects of SIRT1 protein expression and gene polymorphisms rs7895833 on the relationship between hemoglobin levels and physical frailty in elderly adults with comorbid chronic diseases: A moderated mediation analysis." We appreciate the opportunity to clarify and improve our manuscript based on your feedback. Below, we address each of your concerns and describe the amendments we have made to the manuscript accordingly.

 Clarification of SIRT1 Levels (Plasma vs. Cellular):

 We have revised the title and the manuscript to explicitly state that the SIRT1 levels measured and discussed pertain to plasma levels, not cellular protein levels. To provide clarity, we have also added a brief explanation in the Methods section regarding the plasma source of SIRT1 and its relevance to our study's objectives. 

 Genotyping Gel Image Clarification:

 We acknowledge the confusion regarding the genotyping gel image and appreciate your pointing out the discrepancies. We have revised the image to include a clearer representation of the marker bands and accurately labelled each band to correspond with the correct DNA size. 

 Inclusion of Correlation Plots:

 Following your suggestion, we have now included correlation plots that depict the relationship between SIRT1 levels, frailty, and other critical parameters for all 132 participants. These plots provide a visual representation of our findings and strengthen the manuscript. 

 Reduction of Repetitions and Typographical Errors:

 We carefully reviewed the manuscript to eliminate redundant information between the Introduction and Discussion sections. We have also conducted a thorough proofreading to correct typographical errors throughout the text. These changes aim to improve readability and accuracy, and we believe they significantly enhance the manuscript's quality.

 Revision of Conclusions:

 We have streamlined the Conclusions section to succinctly summarize our findings without speculative statements. The revised Conclusions focus on our study's contributions to the field and its implications for future research, avoiding overgeneralization. 

 We hope that these revisions adequately address your concerns and improve the quality and clarity of our manuscript. We are grateful for your constructive feedback and the opportunity to enhance our work. We look forward to your further suggestions and the possibility of our manuscript being accepted for publication.

 Sincerely,

 Dedi Ardinata and co-authors

# Reviewer report: Reviewer response for version 1

**DOI:** 10.5256/f1000research.146512.r243614

**Published:** 2024-02-20

**Authors:** Ramu Manjula

**Affiliations:** 1 Yale University, Connecticut, USA

This study aimed to investigate the relationship between SIRT1 levels, SNP rs7895833, hemoglobin levels, age, and physical frailty (frail score) among 132 older Indonesian adults with comorbid chronic diseases. The present study aligns with recent research suggesting that the association between low hemoglobin levels and physical frailty among older adults with one or more chronic diseases is influenced by age. Furthermore, this study revealed that the SNP genotype AG-GG and high SIRT1 levels further moderated the relationship between low hemoglobin levels and physical frailty in elderly adults with comorbid chronic diseases. While multiple studies support the protective role of increased SIRT1 expression in the body, the study highlights that SNP rs7895833 A>G affects the expression of the SIRT1 gene.

 The manuscript is structured well, and the authors have proved their hypothesis. As mentioned by the authors, along with the sample size the strong limitations of this study are lifestyle habits, environmental factors, and medications, which could have influenced the results.

 I believe that conclusions cannot be drawn based on these results.

Is the work clearly and accurately presented and does it cite the current literature?

Yes

If applicable, is the statistical analysis and its interpretation appropriate?

I cannot comment. A qualified statistician is required.

Are all the source data underlying the results available to ensure full reproducibility?

Yes

Is the study design appropriate and is the work technically sound?

Yes

Are the conclusions drawn adequately supported by the results?

Yes

Are sufficient details of methods and analysis provided to allow replication by others?

Yes

Reviewer Expertise:

Sirtuin biology

I confirm that I have read this submission and believe that I have an appropriate level of expertise to confirm that it is of an acceptable scientific standard.
